# Discursive Interaction and Agency in Transitional Justice: A Conversation Analysis Perspective

**DOI:** 10.1080/17502977.2024.2362002

**Published:** 2024-07-02

**Authors:** Denisa Kostovicova

**Affiliations:** European Institute, London School of Economics and Political Science, London, UK

**Keywords:** Transitional justice, agency, interaction, discourse, communication

## Abstract

The article presents discursive interaction as an analytical framework for capturing agency in transitional justice (TJ). Embedded in the paradigm of Conversation Analysis, which foregrounds the interactive nature of communication, the framework sets out how the exercise of agency is facilitated or constrained at the micro level of talk in conversational sequences. The article shifts attention to the mechanics of interactions in TJ, which are demonstrated empirically with patterns of interactions in the Croatian Parliament. The reconceptualization of agency based on the systematic analysis of how speakers engage with each other advances emerging theories of interactive TJ both conceptually and methodologically.

## Introduction

The question of agency is central to transitional justice (TJ). Efforts to address the legacy of human rights abuse hinge on the ability of those seeking justice and those advocating on their behalf to articulate victims’ needs, influence TJ policy and shape TJ practice. However, formal and informal TJ deliberations and practices often fail to pave the way for either justice or acknowledgement of the victims. These adverse impacts bring to the fore the enduring puzzle of agency in transitional justice, which homes in on its conceptualization and on identifying conditions conducive to its actualization. This article asks: how can victims and their advocates exercise their agency to promote justice?

The article presents discursive interaction as a novel analytical framework for capturing agency in transitional justice at a micro-level of discourse. This framework engages with TJ scholars’ growing interest in micro-level spaces, processes and practices, spurred on by the ‘local turn’ in TJ. The micro level has been understood as a space and a voice belonging to local communities impacted by external peacebuilding interventions, including TJ (Kochanski 2020; Leonardsson and Rudd 2015).^1^ A more granular understanding of the micro level centers on individuals, including interpersonal and intrapersonal, i.e. cognitive, dimensions of their involvement with TJ (Cárdenas, Casas-Casas, and Méndez 2014). The micro-level perspective has made visible overlooked aspects of TJ and elaborated on complexities involved in achieving redress for past wrongs, such as negative micro-level effects of positive macro-level changes (Macdonald 2015) or, conversely, successes at the micro level despite the failure of TJ at the macro level (Gready 2005). The study of individual and social attitudes, experiences and discourses has informed these insights.

Preoccupied with how discourse reflects power(lessness) of TJ actors or shapes TJ norms, scholars have given voice to the silenced and unpacked normative contestations. Despite a substantial body of research about how TJ actors engage with discursive macro-structures, for example, the global TJ discourse, the minutiae of people’s interactions at a micro level of interpersonal interactions have received scant attention. Evrard, Bonifazi, and Destrooper (2021, 435) have called for recognizing ‘the multiple sources of potential power and of power differentials’ in TJ. The structure of conversation in which people are involved when pursuing justice is one such source. The micro level of discourse is a terrain where the power of TJ actors to enact transformative change is constituted as well as constrained, and it remains little understood.

I address this gap in the transitional justice scholarship and develop a concept of discursive interaction that reframes agency as a product of interaction. The elaboration of discursive interaction fits broadly within the study of interaction behaviors (Keyton 2018). The focus on verbal interactions builds on a recently growing interest in the role of communication in transitional justice and, more broadly, the promotion of peace (Nakagawa 2018; Pukallus 2022). It also complements scholars’ long-standing interest in the voice of victims and their advocates in transitional contexts (Gready 2008; McEvoy and McConnachie 2013).

The reconceptualization of agency at a micro level of discursive interaction mobilizes Conversation Analysis, which is a theoretical perspective and a research program. It is preoccupied with the interactional nature of communication and theorizes talk-in-interaction (Atkinson and Drew 1979; Heritage and Clayman 2011). Conversation Analysis directs us to scrutinize patterns of interaction at the micro level of talk. This, in turn, allows us to identify conversational constraints and opportunities that determine actors’ ‘agentic prospects’ (Gibson 2000, 370). The framing of discursive interaction is also informed by relational ontology. It situates agency within a web of relations among actors involved in a verbal exchange, where the nature of ties is constitutive of their power (Gibson 2000; Gnisci 2018). Such a conceptualization of agency in TJ shifts the analysis from effects of exogenous macro-level structures on actors’ ability to realize their interests – the staple of critical TJ research – to hitherto unexplored endogenous structures constituted by patterns of interaction at the micro level of talk.

When we locate agency in discursive interaction, we can observe and evaluate how it is exercised through methodical interrogation of ‘interactional trajectories’ (Voutilainen and Peräkylä 2014) of actors involved both in formal and informal TJ processes. By studying the architecture of talk, we can understand ‘*how* it worked, *what* worked and *what* did not work’ (Stokoe 2018, 3). We can then identify opportunities and obstacles to people’s influence at the micro level of communicative exchange. The framing of discursive interaction and agency therein advances the field in three ways.

It connects with an emerging interest in the interactive dimensions of transitional justice (Ceva and Murphy 2022; Ullrich 2016), which have attributed normative effects to interactions within TJ institutions and to interactions between representatives of these institutions and a range of constituencies with a stake in TJ. The framing of discursive interaction advances these efforts with its conceptual and empirical elaboration of the micro-level mechanics of interaction that produce normative effects both in institutional and informal settings where TJ is practiced. This research takes note of Lundy and McGovern’s proposition (2008, 292) that ‘[t]here is a need to foster agency by thinking imaginatively outside the “prevailing transitional justice box”’. It extends discourse-based explanations of agency in TJ by departing from critical discourse analysis accounts that associate agency with discursive resistance to hegemonic norms and structures (for example, Bernath and Rubli 2016; Gready 2008; Mazzei 2011; Preysing 2016) and from post-structuralist accounts of agency that put emphasis on the relevance of subject positions (Baines 2015; Porter 2016). The discursive interaction framework focuses the analysis on the relational nature of agency embedded in the network of verbal exchanges. Lastly, by leveraging Conversation Analysis, with its interest in talk-in-interaction, this article contributes to efforts to produce new empirical evidence for normative claims about TJ through methodological innovation (Olsen, Payne, and Reiter 2010; van der Merwe, Chelin, and Ndinga-Kanga 2022).

The development of the conceptual framework of discursive interaction and of agency as a product of interaction in the ensuing sections of this article is followed by an empirical demonstration of discursive interaction in the Croatian Parliament. It demonstrates interactional opportunities for female members of parliament (MPs) to exercise their agency when asking oral parliamentary questions about TJ in the Croatian Parliament, along with constraints they encounter.

## Interactions in transitional justice: Hidden in plain sight

The notion of interaction has always been implicit in scholarly discussions about TJ. Reckoning with the legacy of human rights abuse committed during conflict or under repressive rule is by its nature an interactive process. Justice-seeking implies an interaction with something, such as ideology, or someone, such as a prosecutor examining a witness. However, little effort has been made to theorize interactions themselves. To paraphrase Heritage (2001, 4), TJ scholars have frequently made reference to interaction but ‘have had little to say how interaction works, treating it as an invisible and inscrutable “black box”’.

Scholars have fallen back on focusing on the interactants – the participants in an interaction – at the expense of analyzing their interactions. As a result, the multi-disciplinary TJ scholarship abounds with references to different kinds of interactions. This dovetails with the rapidly broadening scope of TJ. However, in a recent development, scholars have put forth concepts of interactional or interactive justice (Ceva and Murphy 2022; Ullrich 2016). Although they have foregrounded interactions and focused on their normative implications, the understanding of the mechanics of interaction(s) that lead to those normative outcomes still remains scant.

The idea of interaction in TJ was first introduced through discussions querying the relationship between transitional justice and peace, understood broadly. Growing awareness of political, social and economic aspects of transition from war to peace has raised questions about how TJ practices interact with specific dimensions of transition, such as democratization; (re)construction of the rule of law; human rights; and development (McAuliffe 2013); as well as about the mediating role of different factors, such as education, the media and new technologies, to name a few. At the same time, responding to the proliferation of TJ practices, scholars have investigated how different TJ instruments, such as trials and truth commissions, interact with each other (Gready and Robins 2020; Olsen, Payne, and Reiter 2010). They have also considered their spatial dimension as manifested in interactions of global, national and local transitional justice processes and actors (Ainley, Friedman, and Mahony 2016; Arnould 2016; Selim 2018). All along, they have examined a plethora of TJ actors, ranging from legal practitioners, commissioners, victims and perpetrators, to politicians, civil society activists, artists and their engagement in TJ processes. More recently, Ullrich (2016) and Ceva and Murphy (2022) have presented attempts to conceptualize the interactive aspect of TJ.

In Ullrich’s (2016) view, interactional justice is constituted through institutional discourse, everyday practices and communicative interactions among practitioners. Her theory illuminates ‘how ideas of “local” and “global” are constructed and then deliberately conflated in the production of international criminal justice for victims’ (Ullrich 2016, 546), as illustrated with a study of local intermediaries of the International Criminal Court in Kenya and Uganda. Ceva and Murphy’s (2022) concept of interactive transitional justice is more ambitious than Ullrich’s (2016). It draws on Ceva’s (2016; Ceva and Murphy 2022, 763) elaboration of interactive justice and interrogates how institutional processes ‘may structure a just form of human interaction.’ They contend that ‘transitional justice processes may be evaluated in virtue of the qualities of the relationship dynamics they themselves cause or constitute’ (Ceva and Murphy 2022, 763) and by the extent to which this relationship dynamic is associated with broader societal relational transformation. For example, they cite a decision not to hold a second plebiscite on the revised Final Agreement prior to its ratification by the Colombian Congress in November 2016 as an ‘interactive *injustice*’ in the case of Colombia (Ceva and Murphy 2022, 772).

While both accounts of the interactive nature of transitional justice depart from other cursory references to interactions, they are limited by a ‘weak’ relational approach to transitional justice (Slife 2004, 159). Neither Ullrich’s (2016) account of the dynamics of normative contestation, nor Ceva and Murphy’s (2022) account of relationship dynamics, consider how the power of participants in TJ processes is constituted by their relations. A strong relational approach informed by a relational ontology conceives of actors as ‘actors-in-relations’ (Qin 2016, 36; Slife 2004, 159). From this perspective, actors’ power is determined by the structure of their ties and connections to other actors rather than by actors’ attributes, such as victimization, ethnicity, race, gender, etc. (Jackson and Nexon 2019, 583). Despite Ullrich’s (2016) and Ceva and Murphy’s (2022) focus on the interactive nature of TJ, they do not explain *how* the relationship transformation is achieved *interactionally* in view of actors’ relations and what the mechanics of interaction imply for agency of those involved.

The framing of discursive interaction follows Ullrich (2016) and Ceva and Murphy (2022) in foregrounding an interactive nature of TJ in its conceptualization. Embracing the relational ontology innate to interactions, the framing of discursive interaction pairs a discourse-focused conceptualization of interaction with guidance for its systematic empirical analysis and evaluation. By doing so, it demonstrates how agency is ‘done’ in a micro-interactional environment of verbal exchanges.

## Agency in transitional justice: A normative solution and an analytical challenge

The question of justice is inextricably linked to the ability of actors in TJ to realize their agency. The concept of agency has become both a heuristic and a benchmark for the normative evaluation of TJ practices. Although TJ scholars refer to ‘agency’ frequently, they seldom define it. As a consequence, agency has many meanings, some of which are overlapping. A review of different takes on agency in TJ, presented as follows, relates the interactional production of agency at a micro level of discourse to TJ scholars’ preoccupation with structure and power in their discussions of agency.

The concept of agency in the TJ scholarship is often associated with control by local actors. As such, agency constitutes a dimension of local ownership (Sharp 2018). While agency grants visibility to marginalized actors in TJ, it should not be conflated with their representation (Hudson 2012) or recognition (Menzel 2020). As Gready (2008, 47) spells out, it is important to ‘nudge empowerment beyond the lone voice to ownership and control of agency’. Likewise, agency is distinct from action (Björkdahl and Gusic 2015). Emirbeyer and Mische (1998, 1004) originally refined the distinction between actor, agent and agency, specifying that ‘agency itself remains a dimension that is present in (but conceptually distinct from) all empirical instances of human action; hence there are no concrete agents, but only actors who engage agentically with their structuring environments’. From this perspective, agency presumes ability or power to enact transformation (Creary and Byrne 2014, 68).

Above all, the concept of agency as applied in transitional justice is value-laden. For example, Murphy (2017) qualifies agency in TJ as ‘moral agency’ of the victims. Agency in TJ is defined by an ability to achieve goals that people value. It departs from a purely rationalist interpretation of agency defined by ‘goal seeking and purposivity’ (Emirbayer and Mische 1998, 963). The value aspect of agency as it has been deployed in TJ is best understood from Sen’s (1985) perspective on agency. Sen (1985, 204) sees people as being subject to ‘the moral accounting by others not only as people whose well-being demands concern, but also as people whose responsible agency must be recognized’. Unlike its moral dimension, the question of intentionality remains a moot point in these discussions about agency in the TJ scholarship. Arguments such as Menzel’s (2020), which posits that agency is defined by the existence of motivation for change, stand alongside alternative perspectives that locate agency in the domain of the everyday. In these alternative conceptions of agency, a change, which is political and normative, can arise from actors’ apolitical, unpremeditated and value-free action.

Because it is imbued with value, agency has been overwhelmingly theorized by TJ scholars from the vantage point of the victims of human rights abuse. This research agenda rests on two assumptions: firstly, that those who were harmed by violence are most in need of justice and hence should have a say in it, and, secondly, that TJ processes may be only nominally sensitive to the needs of victims and survivors; without meaningfully centralizing victims and survivors’ needs, TJ processes risk disempowering those they seek to support (Nyseth Brehm and Golden 2017, 105–106). Consequently, the extent to which victims are able to bring about change that is in line with their needs is indicative of their agency and of the quality of transitional justice. Such evaluation takes account of a paradox: the very act of inclusion of victims in TJ processes can simultaneously imply their disempowerment and de-agentification, for example, through gendered dynamics of inclusion and exclusion (Björkdahl and Mannergren Selimovic 2015, 170; cf. Skjelsbæk 2012; Schulz 2020, 131–159) or by treating some victims as more deserving of justice than others in the context of so-called victim hierarchies (Andrieu 2010, 543–544; Barton-Hronešová 2020). This has brought to the fore complexities of victims’ ability to realize their agency and achieve justice for all.

Victim-centered approaches have generated nuanced insights into how victims fare within the model of transitional justice (Nyseth Brehm and Golden 2017, 105–106) and how they are able to realize their agency. However, Macdonald’s (2015) meta-analysis of the local turn in TJ has shown that the scholarship has also been restricted by virtue of its preoccupation with victims at the expense of other actors. This is especially the case when conceptualizing agency. The concept of agency presumes action that advances one’s values. But, as Sen argues (1985), agency can also be other-regarding, in the sense of being oriented to advancing goals that matter to another person or group (Alkire 2008, 5). In other words, the actions of many other actors working for justice for the victims, such as politicians, artists, curators, teachers, journalists and human rights activists morally invested in struggles for justice,^2^ have been studied without being brought systematically into theoretical discussion about the meaning of agency in TJ.

In a parallel development, TJ scholars have begun to criticize explanations focused exclusively on agency. Hoddy and Gready (2020, 562) have cited the need to ‘render transparent’ the structural and relational contexts within which the enactment of agency is constrained. According to them, these wider systems and structures that are root causes of human rights violations need to be challenged rather than treated ‘as “inert” background and context for interventions’ (Hoddy and Gready 2020, 572). Similarly, Menzel’s (2020, 604), conceptualization of agency as situational has called for a careful analysis of ‘specific contexts in which “locals” (or any other actors) may have agency – or not’ and for scrutiny of ‘actors’ doings in those contexts’ (cf. Skjelsbæk 2012; Tamayo Gomez 2022, 55). These critiques highlight both the macro– and microstructures that enable or constrain agency. As such, they call for better understanding of ‘actors’ relative power’ (Christopoulos 2006, 761). If we locate agency within discursive interactions, we need to conceive of power as relational, arising from connections held with other actors (Hafner-Burton, Kahler, and Montgomery 2009, 560; Jackson and Nexon 2019, 589), while at the same time considering the conversational constraints (Gibson 2000). The next section sets out an approach that conceives the interactional production of agency at the micro level of a communicative exchange.

## Discursive interaction from a conversation analysis perspective

A family of social interactional approaches to discourse can inform a novel conceptualization of agency and evaluation of prospects for its realization in TJ processes. Common to these approaches is the premise that social interaction is ‘the basis of social order’ (van Dijk 2014, 10). From a social interactional viewpoint, structures are ‘actualised in interaction,’ which means ‘that they are inescapably influenced by interaction and so constantly vulnerable to innovation and potential change’ (Jaspers 2013, 140). Hence, new opportunities for and obstacles to justice for the victims can be identified by scrutinizing the structures of discursive interactions.

Discursive interaction is about ‘language in use’ (Gee 2014, 8), which is an overarching definition of discourse. Its specific meaning in the discursive interaction framework is that of Holzscheiter’s (2014, 146) notion of a micro-level ‘communicative exchange.’ Leveraging the Conversation Analysis perspective will allow us to identify and evaluate previously overlooked ‘agentic prospects’ (Gibson 2000, 370), which actors have in TJ processes when they communicate with each other.

As a conceptual paradigm and as a mode of analysis, Conversation Analysis (CA) ‘placed the empirical study of communication at the heart of social science enterprise’ (Wooffitt 2005, 5). Rather than explaining social action as a consequence of norms (in terms of what is allowed and what is sanctioned) or in terms of strategies in pursuit of actualization of interests, CA views social action as a response to ‘moment-to-moment contingencies’ of human interaction (Pomerantz 2021, 3).^3^ CA’s specific concern is with actions performed by language (Wooffitt 2005, 10). Wilkinson (2006, 56) specifies that the talk is produced to ‘*do* something: to corroborate, to challenge, to boast, to tease, to emphasize our suffering (or to downplay it), and so on.’ When CA scholars state that they are interested in what people are doing, they are expressing an interest in what people are saying or not saying, the particular manner in which they are doing it, the particular time of their utterance within an interaction and what is thus achieved (ten Have 2007, 16). The discursive interaction framework is rooted in investigations of an interactional order and a methodical scrutiny of ‘the organisation of talk as joint activity and as communication’ (Wetherell 2001, 5).

All conversations, including those concerning redress for human rights abuse, are ‘composed of myriad of interactional events taking place among specific people at specific times and places’ (Gee 2014, 72). A CA perspective overcomes descriptive references to interactions in transitional justice and paves the way for the systematic, micro-level analysis of talk-in-interaction. It allows us to go beyond a well-trodden analytical path in the transitional justice scholarship, which approaches actors’ discourses as static constructs determined by who utters them, for example, victims or politicians, and by their location in their political, social and historical contexts. CA is concerned with the ‘organisation of conduct *within interactions*’ (Clayman and Teas Gill 2012, 120). It turns on the ‘sequential organisation’ of talk, which refers to ‘any kind of organization which concerns the relative positioning of utterances or actions’ (Schegloff 2007, 2). The stress here is on relative, which demonstrates the dependence of actions as manifested in language; for example, an apology will be followed by acceptance (or refusal). Such dependence is captured in Drew’s (2013, 132) definition of verbal interaction as ‘the contingently connected sequences of turns in which we “act”, and in which the other’s – our recipient’s – response to our turn relies upon, and embodies, his/her understanding of what we were doing and we meant to convey in our (prior) turn.’

Insofar as it aims to discern patterns of discourse, like other types of discourse analysis, CA’s distinct concern is with ‘orderliness’ of talk (Psathas 1995, 2; Sidnell 2010). This idea presumes that ‘social interaction is orderly on an individual action-by-action, case-by-case level’ and reverses the old social science perspective that social interaction is disorderly (Heritage 2001, 52). The idea of order implies that all instances of naturally occurring conversation are ‘equally explicable in terms of general rules and procedures’ (Gibson 2012, 12–13). This makes the establishment of the patterns of talk through interactions analytically rewarding, while violations of those rules and procedures that govern orderly talk become analytically puzzling. For example, consider the one-speaker rule (Schegloff 2007, 1). The general rule is that people do not talk over each other, which makes overlapping talk, as a violation of that rule, an issue of particular interest in the field of Conversation Analysis. Detecting interactional patterns can provide a new insight into agency and its actualization.

## Conversation analysis and agency as a product of interaction

Understanding of conversational rules has a direct bearing on how we can conceptualize agency in TJ from a CA perspective. Bringing the issue of agency into CA, Gibson (2000) has formulated the phenomenon of conversational agency. Conversation Analysis scholars are concerned with interactional constraints: they study how a speaker is constrained by what an interactant said in the previous speaking turn. To illustrate: if a *yes-or-no* question is asked, a ‘*yes*’ or ‘*no*’ is expected to be an answer. However, Gibson (2000, 376) highlights ‘looseness’ in interactional constraints, which reflects the choices that speakers have. In other words, interactional constraints exist, but they do not necessarily predetermine the sequence of conversation. As he puts it, ‘looseness lengthens the chains, but does not release its manacles’ (Gibson 2000, 376). To continue with the illustration above: a speaker may decide to avoid providing a ‘*yes*’ or ‘*no*’ as an answer – which is a common strategy of evasion. Accordingly, understood from a CA perspective, agency is defined as ‘action that furthers an actor’s idiosyncratic ambitions in the face of localized constraints that otherwise suppress the very same’ (Gibson 2000, 380–381). But, conversational latitude and agential prospects are also shaped by relational considerations.

Gibson (2005) has brought into dialogue conversation-analytic and network-analytic approaches, with their distinct accounts of social interaction. The micro-level approach of Conversation Analysis is concerned with the sequential organization of talk, while network analysis is concerned with networks as meso-level relational structures. According to Gibson (2000; 2005), an actor’s network position can loosen conversational constraints, which arise from the sequential organization of talk, but it can also impose limitations on what an agent can or cannot do. For example, for members of a team to affirm and build on one another’s remarks, they need to be able to speak in a string of speaking turns; this also implies that others may not cede their speaking turn for a team to achieve their goal pursued in a conversation (Gibson 2000, 377). The argument of network effects on conversation is born out with evidence that conversational norms are modified incrementally in light of relational commitments and actors’ relational power (Gibson 2005). A finding from Gibson’s (2005, 1588) analysis about how the consideration of power comes into play in the conversational structure is particularly insightful for TJ, considering the field’s concern with power hierarchies. Analyzing a network of interactions, he found that subordinates were not only more likely to turn the floor back over to their superiors, but were also ‘particularly *averse* to directing the floor away from them’ (Gibson 2005, 1588). This finding has significant implications. In addition to identifying empirically an interactional pattern that people are more likely to turn the floor over to someone more powerful rather than to their equals, the pattern also reveals a role ‘dutiful subordinates’ may be expected to play in amplifying a superior’s remarks (Gibson 2005, 1588).

Drawing on Gibson (2000; 2005), a CA perspective on discursive interaction within which agency is produced accommodates a relational ontology. Conceptually, discursive interactions are defined by the sequential nature of communicative exchanges, while cognizant of their relational structure. The interactional production of agency has empirical implications for specifying the unit of analysis and the data.

From a CA perspective, the manner in which a certain practice is deployed and is responded to represents the analytic resource (Heritage 2013, 129). Hence, a CA scholar is interested in how a speaker designs a turn-at-talk (Drew 2013, 132). Therefore, the empirical investigation focuses on turn-taking as the unit of analysis. At the same time, Conversation Analysis is concerned with everyday talk, understood as naturally occurring talk in face-to-face or in synchronous or asynchronous computer-mediated communication and on social media, such as X (formerly Twitter). The talk may take place in formal, i.e. institutional, and informal settings (such as courtrooms, parliaments and schools, as well as among family and friends). It is of critical importance to CA researchers that the data is not artificial in a sense of it being induced or manipulated, as in research interviews or laboratories. This requirement stems from the understanding of ‘talk-in-interaction as a “situated” achievement’ (ten Have 2007, 9).^4^

Scholars have only started to exploit the analytical potential of a Conversation Analysis paradigm in politics and international relations (Gibson 2012; Heritage and Clayman 2013; Whitehead 2012). Of particular relevance to scholars interested in TJ is that CA has enhanced our understanding of how people address delicate issues, such as rape, HIV/AIDS or a cancer diagnosis (Drew 1992; Jefferson 2015; Silverman and Peräkylä 1990). Conversation Analysis can lead to the discovery of systematic patterns of order in discursive interactions in TJ and enhance our understanding of how agency is enacted interactionally. As Heritage (2009, 312) puts it, ‘a powerful sense of injustice can be mobilized by departures of the conventions of the interaction order’. Conversely, we can discover how agency is exercised by scrutinizing interactional patterns and identifying opportunities for asserting a voice at the micro level of a communicative exchange. The following section provides an empirical demonstration of how agency can arise in interactions between female and male MPs deliberating on TJ policy in the Croatian Parliament.

## Discursive interaction and agency: An empirical demonstration

To demonstrate how we can locate and identify agency at the micro level of discursive interaction, I evaluate women’s voice in deliberations of the TJ policy in the Croatian Parliament. This demonstration engages the debates on gendered TJ, which overlooks or sidelines women’s justice needs related to their experience of violence that is different from men’s (Weber 2022). Existing scholarship on impediments to gender-just peace has highlighted masculine norms and structures that impede the recognition of harms suffered by women in advocacy, practice and outcomes of a variety of post-conflict justice practices (Campbell 2022; Swaine 2018). These norms and structures are underpinned by macro-level discourses operating at a global, national and local levels. Hence, degendering TJ starts with efforts to enable women’s access to peace negotiations and other fora where TJ issues are discussed (for example, domestic and international institutions) and to ensure their equal representation in TJ instruments (for example, as judges in war crimes trials).

It has been assumed that women’s presence in these processes will bring opportunities for women to articulate their justice needs and to shape TJ policies and practices accordingly (Rooney 2007). However, practitioners and scholars have recently begun to highlight the continued marginalization of women and their concerns even when they participate alongside men in various processes related to TJ (UN Women 2022). This is known as a problem of women’s presence without influence (Cahn and Ní Aoláin 2009; Castillo Diaz and Tordjman 2012). When we restate the problem of women’s marginalization in TJ in this way, we highlight the limited power of macro-level discourses that underpin masculine norms and structures to fully explain gendered justice. This problem points to a need to better grasp interactional constraints operating at a micro level of discourse to evaluate when and how women can express their concerns.

Leveraging Conversation Analysis to capture interactional patterns and agency at a micro level of discourse builds on sociolinguistic studies of gender-differentiated language that posit that men and women speak differently. These differences become salient in mixed-sex interactions in a variety of settings: in informal interactions, such as small groups, and in institutions, such as parliaments (Holmes 1995; Walsh 2001; Shaw 2020). Arguably, they will also be observed during the parliamentary question time in the Croatian Parliament. Since the end of the 1991–5 Croat–Serb conflict, Croatia has been dealing with post-conflict justice issues. As a national legislative body, the parliament has played a seminal role in steering the country’s TJ policy, which has been widely criticized for being gendered and ethnocentric (Banjeglav 2012; Pavlakovic 2010; Sokolić 2019).^5^ Of primary interest here is how women have participated in the parliamentary speech in order to influence the TJ policy, including advocating for women’s TJ needs.

Parliamentary questions involve an exchange between the asker and the recipient. They represent one form of interaction. As Hayano (2012, 396) argues, ‘questions are a powerful tool to control interactions’ through, for example, pressuring answerers for a response or imposing an agenda. Scholars have studied women’s and men’s speaking style in parliaments, which focuses on *how* meaning is expressed (Tannen 1994, 192). Distinguishing between men’s aggressive and adversarial language and women’s polite or civil language, they have identified gendered speaking behavior in parliaments (Ilie 2013; Shaw 2020; Walsh 2001). Men’s adversarial language has broader consequences. It contributes to constituting parliaments as masculine institutions and marginalizes women and their interests. Leveraging CA to parliamentary speech about TJ, we can capture women’s agency by examining two dimensions of interaction: (1) the style of women’s questions and the extent to which they exert pressure on the recipients of those questions, and (2) considering gender of the recipients of those questions to glean the nature of connections established through the communication exchange.

I use an original dataset consisting of 390 oral questions that members of parliament (MPs) in Croatia asked about various aspects of the country’s TJ policy from 2004–20. The dataset was compiled by using transcripts of the parliamentary questions, available on the official website of the Croatian parliament (Sabor).^6^ Questions about TJ were first identified using TJ-related search terms combining a deductive and inductive approach. Then, a qualitative human coding informed by CA was conducted of two stylistic features of parliamentary questions: polarity and accountability.^7^ Following Clayman et al. (2006, 569), these codes capture the interactional dimensions of parliamentary questions, combining formal linguistic features of question design and the content of questions – both of which put pressure on the recipients of those questions.

Considering that questions have recurrent grammatical features (Harris 2001; cf. Fenton-Smith 2008, 99), polarity is operationalized as a *yes-or-no* question through the linguistic form, which invites a *yes-or-no*-type response (Clayman et al. 2006, 567). In the context of a parliamentary question time, polarity expresses a form of pressure on the answerer because it limits acceptable types of an answer and reduces the scope for evading an answer (Shaw 2020, 64). A *yes-or-no* question design limits resources available to a recipient, while question design is used to accomplish specific institutional goals and outcomes (Raymond 2003, 956–957), such as accounting for a missing or delayed policy and action.

Turning to the content of questions, accountability is operationalized as an explicit request to the recipient to justify government policies. As Clayman et al. (2006, 31) point out, questions demanding accountability are to an extent aggressive and accusatory, since they ‘decline to accept policy at face value’. As such, they are indicated by the occurrence of *how could you*- and *why did you*-type of questions (Ibid.). This form of questioning also serves as a mechanism of agenda-setting. They impose a topical agenda (in terms of what is being talked about) and restrict the recipient in setting his or her own agenda (Hayano 2012, 402).

The patterns of interaction will be derived from a scrutiny of micro-level exchanges between men and women. They will reveal how women counter men’s domination in parliaments, perpetuated through men’s numerical overrepresentation and adversarial discourse, itself associated with men’s language. Scholars have argued that aggressiveness, adversariness and sexism in parliaments alienate women and reduce their participation in policy deliberation, whereas women’s polite and cooperative language undermines their effectiveness as policymakers (Ilie 2018, 600; Shaw 2020).

Before proceeding to evaluate the interactional features of women’s and men’s parliamentary questions, the pattern of women’s participation in the question-asking activity in the Croatian Parliament is presented. As shown in Table 1, women ask fewer parliamentary questions than men; that is, 17.43% questions are asked by women compared to 82.56% questions asked by men.^8^ Considering that historically women have comprised around 20% of all members of parliament (Poljak 2022), we observe a degree of proportional underrepresentation of women’s parliamentary questions among all parliamentary questions.^9^

**Table 1.** Descriptive statistics of the number of parliamentary questions by gender.

	Questions count	Questions percentage
Asked by men	322	82.56%
Asked by women	68	17.43%
	**390**	**100**.**00%**

**Figure 1.**
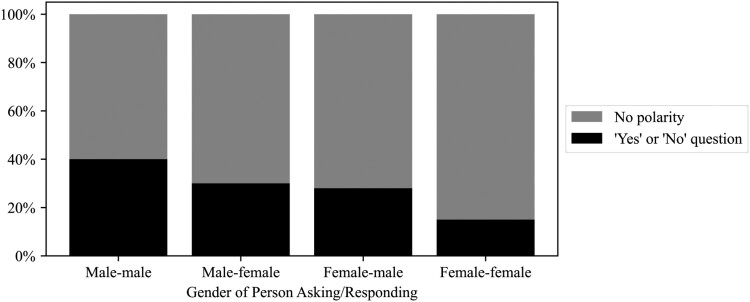
Percentage of questions by polarity.

When we examine the results of the analysis of polarity, whereby polar, i.e. *yes-or-no,* questions put more pressure on the recipient than questions with other linguistic features, we can observe that male MPs ask more polar questions as a proportion of all questions they ask than female MPs. 37% of men’s questions are polar as opposed to 21% of women’s questions.^10^ However, important nuances come to the fore when we investigate interactions by gender. As shown in Figure 1, there is a notable difference in the proportion of polar questions out of all questions women address to men (28%) compared to the proportion of polar questions out of all questions women address to women (15%). We observe that male MPs address proportionally more polar questions to men than to women, i.e. 40% as compared to 30%.^11^

**Figure 2.**
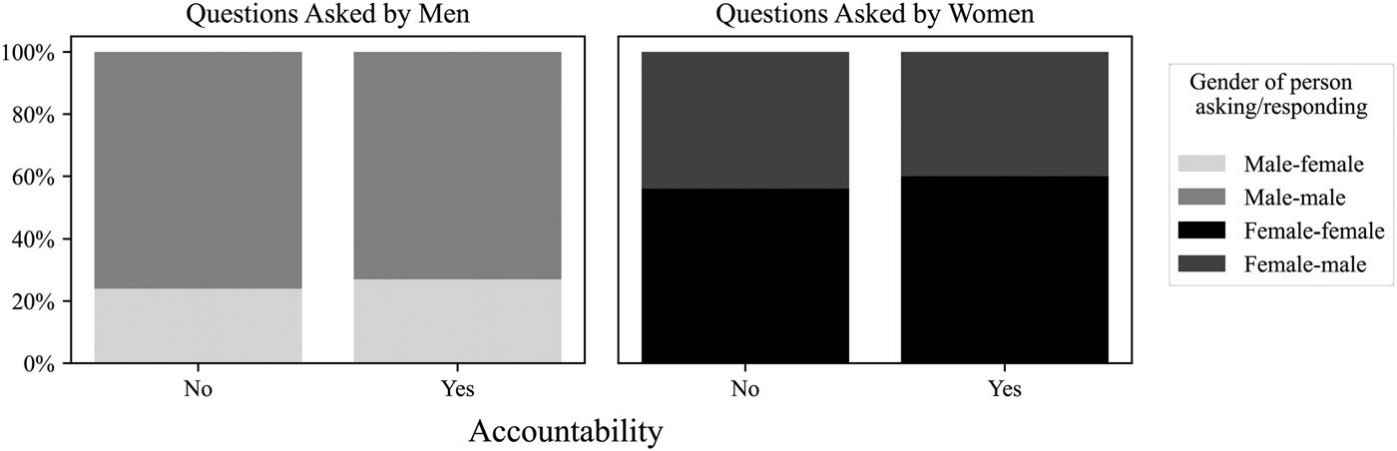
Percentage of questions by accountability and gender of the asker.

Lastly, when we investigate the content of the questions to analyze requests for accountability as another aspect of interaction, we observe that the proportion of men’s questions demanding accountability of the recipient (as opposed to those questions that do not) is almost the same as women’s, i.e. 30% and 29%.^12^ When we consider the gender of the asker and the recipient, as shown in Figure 2, of all women’s questions with accountability requests, 60% are addressed to female ministers and 40% to male ministers. This stands in contrast to the proportions of all men’s questions with accountability requests, of which 73% are addressed to men and 27% to women.

A granular analysis of talk-in-interaction evidenced in parliamentary questions about TJ in the Croatian Parliament reveals macro-level masculine domination and micro-level opportunities that female MPs seize to assert their agency. Men ask more parliamentary questions overall, which aligns with their numerical domination of the parliament. This form of dominance is reinforced through an assertive, masculine style of questioning, evidenced in some but not all interactional features and patterns of their questioning. For example, men set the tone with polar questions. However, women are as assertive as men when we assess how many of their questions contain a demand for accountability as compared to men. These interactional patterns are further refined when we consider to whom men and women address their questions. Although overall women use an assertive linguistic style of questioning, i.e. polarity, less than men, they are more assertive in female–male than in female–female interactions. This indicates women’s attempt to counter men’s dominance at a micro level of a communicative exchange through ‘linguistic participation’ (Shaw 2020, 47). But, this is not the case with accountability (derived from analyzing the content of their questions), since women press female ministers to be accountable more than male ministers. At the same time, we observe that male–male interactions are a micro site where all masculine features of parliamentary interaction examined in this study are entrenched.

This empirical demonstration of discursive interaction, informed by Conversation Analysis, shows how the TJ policy in parliament is interactionally ‘produced, managed, recognised and used’ by co-participants (Atkinson and Drew 1979, 20). Women’s agency comes into full view when, following (Emirbayer 1997, 294), we scrutinize ‘concrete transactions within relational contexts’, such as the context of an ‘ongoing conversation’. From the CA perspective, women’s exercise of their agency, as manifested in the style of questioning, is embedded in the web of relations involving men and women woven by their verbal exchanges and observable at the level of turn-taking in the parliament. We can identify interactional patterns that constitute endogenous structures, within which women create opportunities to resist masculine dominance at a micro level of conversational exchange, alongside those opportunities they forfeit.

This empirical demonstration that locates agency in micro-level discursive interaction can be enriched in two ways. First, additional insights can be gained through an examination of the normative content of parliamentary questions and specific positions on TJ that women advocate, for example, by applying Critical Discourse Analysis or Content Analysis methods (for example, Kostovicova and Popovski 2023).^13^ Secondly, wider interactional patterns can be gleaned by applying probabilistic statistical analysis to capture the relational structure framing women’s agency (Kasper and Wagner 2014). As Emirbeyer and Mische (1998, 963) note, the full complexity of agency should not and cannot not be reduced to a one-sided conception. This empirical demonstration of women’s agency as a product of interaction from the CA perspective adds complexity to existing accounts of agency that rely on other discourse analysis methods in the field of transitional justice. It also presents new possibilities for their further integration as well as for other forms of method mixing. Women’s agency observed at a micro level of talk is not a panacea for overcoming all macro-structural forms of marginalization, and continued efforts aimed at women’s equal representation are needed. However, this research shows that macro-level change can sometimes start at a micro level, which is why it is important to understand how.

## Conclusion

This article has presented discursive interaction as a novel framework for conceptualizing and analyzing agency by opening the black box of interaction in TJ. Following Atkinson and Drew (1979, 28), it has made the phenomenon of verbal interaction(s) in TJ ‘the central topic of analysis.’ Grounding discursive interaction in the paradigm of Conversation Analysis has provided ‘a complex, subtle, and repeatable analytic take on the way people coordinate with another’ in verbal interaction (Potter 2010, 670) that reveals, conceptually and empirically, the interactional production of agency. The discourse interaction framework, which presumes a systematic examination of people’s ‘orientation to talk and to each other’ to grasp how social order is produced and reproduced (Gee 2014, 225; cf. Clayman and Teas Gill 2012, 131), provides new insight into how TJ is achieved or stymied. It thus contributes to advancing the study of the interactional nature of transitional justice (Ceva and Murphy 2022; Ullrich 2016).

With its embrace of relational ontology, the discursive interaction framework shows how power and agency can be reconceptualized in TJ. Actors’ power is constituted by their ties to other actors with whom they interact – an insight that has been overlooked theoretically, methodologically and empirically in the existing TJ scholarship. Accordingly, we can conceive of an interactional production of agency and demonstrate it by detecting interactional patterns at the micro level of discourse. Relational sensibility can also downplay and reproduce the practices of hierarchization (Randazzo 2021, 153). Discursive interaction, therefore, also reveals how the exercise of agency can be constrained at the micro level of verbal exchange. By the same token, the framework provides new pointers for addressing disempowerment.

The study of interactions in TJ presented in this article should be understood as just *one* analytical framework to be applied in the field of TJ, more broadly, and in the study of interactions in TJ, more specifically. Focused on the micro level of discourse, it enriches the understanding of other micro-level dynamics focused on the everyday or bottom-up processes TJ processes, understood as informal processes (Kochanski 2020). Notably, the discursive interaction framework can be applied to micro-level verbal exchanges both in formal institutional settings as well as in informal processes and everyday situations,^14^ and to a whole range of actors (such as victims, human rights activists and politicians) to evaluate their agency in the context of TJ. This framework also charts new ways for assessing macro-level consequences of micro-level interactions. Drew (2013, 20) contends that ‘specific language practices are associated with social outcomes, thus enabling the application of language and interaction analysis to real world functions and dysfunctions’, such as the workings of TJ and its varied normative effects. TJ requires change at multiple levels, micro, meso and macro level (Cárdenas, Casas-Casas, and Méndez 2014, 37; Gready and Robins 2020, 286). The elaboration of the micro-level discursive interaction framework advances our understanding of how change at different levels can be connected. It homes in on the mechanics of interaction to explain how transformation is enacted at a micro level with possible wider consequences, because a ‘simple interaction between individuals or groups’ does not in itself bring about change, for example, in social discourse (Mazzei 2011, 437). Lastly, in view of further analytic potential of relational approaches to TJ, the empirical demonstration of discursive interaction points to the necessity of collecting and explaining ‘relational information’ (Escudero, Lee, and Friedlander 2018, 61).

Ultimately, this article connects with ongoing efforts in this scholarship to refine analytical perspectives and methodological tools to more robustly answer questions about the operation and effects of transitional justice (van der Merwe, Chelin, and Ndinga-Kanga 2022). The exercise of agency in TJ, which is complex and multifaceted whilst enacted in vastly different contexts and through different instruments, defies a single explanation of its effects, whether positive or negative (Buckley-Zistel et al. 2013). Nonetheless, our understanding of the field’s moral dictum that past abuse must be addressed to chart an ethical path forward in post-conflict societies depends on a concerted effort to match new approaches and their ontologies with new methods in order to provide new insights.

## Notes

1 I focus on post-conflict transitional justice, although the argument is applicable to TJ in post-authoritarian societies.

2 For example, NGOs (Zvobgo 2020); although not all NGOs will make a positive contribution to TJ (Hellmüller 2012).

3 On development of Conversation Analysis, see Heritage (2001).

4 However, some methods, such as focus groups, generate data particularly suited to Conversation Analysis (Jarvis 2011, 292).

5 Croatia’s TJ policy has been gendered, having prioritized male victims, specifically veterans, at the expense of female victims of wartime sexual violence (Clark 2016; Subotić and Zaharijević 2017).

6 https://www.sabor.hr/. The number of questions includes questions asked within one speaking turn, i.e. sub-questions, in order to capture comprehensively the richness of the questioning activity (Högenauer 2017, 196).

7 All questions were coded independently by two coders at the acceptable level of inter-coder reliability. The results of the Cohen's kappa coefficient (κ) for polarity and accountability are 0.86 and 0.85 respectively.

8 Put differently, a male MP has asked on average 3.5 questions (that include sub-questions) as compared to 2.83 questions asked by a female MP.

9 Table 1 is comprised of all questions asked within MPs’ speaking turns.

10 See Table A1 in the Appendix.

11 Put differently, of all polar questions women ask, 57% are addressed to men and 43% to women.

12 See Table A2 in the Appendix.

13 Pomerantz (2021, 3) allows for the possibility that a speaker’s action in an interaction with another speaker reflects normative constraints rather than being a response only to an issue she or he faces at a particular interactional juncture.

14 For example, see Stokoe (2018) for an analysis of everyday conversations.

## Appendix

**Table A1.** Distribution of men’s and women’s questions by polarity.

	Men frequency and percent	Women frequency and percent
Polar questions	120 (37%)	14 (21%)
Not polar	202 (63%)	54 (79%)
	100%	100%

**Table A2.** Distribution of accountability requests in men’s and women’s questions.

	Men frequency and percent	Women frequency and percent
Yes accountability	98 (30%)	20 (29%)
No accountability	224 (70%)	48 (71%)
	100%	100%
